# Elevated Relative Levels of the C-3 Epimer of 25-Hydroxyvitamin D in Patients with Cirrhosis

**DOI:** 10.3390/nu18071071

**Published:** 2026-03-27

**Authors:** Caroline S. Stokes, Matthias C. Reichert, Pascal Schorr, Frank Grünhage, Dietrich A. Volmer, Frank Lammert

**Affiliations:** 1 Food and Health Research Group, Faculty of Life Sciences, Humboldt University Berlin, 14195 Berlin, Germany; 2 Department of Medicine II, Saarland University Medical Center, Saarland University, 66421 Homburg, Germany; 3 Department of Chemistry, Humboldt University Berlin, 12489 Berlin, Germany; 4 Center for Health Economics Research Hannover (CHERH), Hannover Medical School (MHH), 30625 Hannover, Germany

**Keywords:** Child–Pugh, LC-MS/MS, liver disease, mass spectrometry, steroid hormone

## Abstract

**Background**: Elevated levels of the C-3 epimer (3-epi-25(OH)D) of 25-hydroxyvitamin D (25(OH)D) have been identified in premature infants as compared to most adults, and an immature liver has been suggested as a possible cause. We hypothesised that patients with cirrhosis might present with elevated C-3 epimerisation due to impaired liver function. The aim was to assess whether 3-epi-25(OH)D levels differ in patients with chronic liver disease with cirrhosis vs. those without cirrhosis. **Methods**: A total of 309 patients were included (254 patients with cirrhosis vs. 55 without cirrhosis). Serum 25(OH)D and 3-epi-25(OH)D levels were determined using liquid chromatography-tandem mass spectrometry (LC-MS/MS). **Results**: Patients with cirrhosis had significantly higher median relative 3-epi-25(OH)D concentrations, as compared to patients without cirrhosis (7.4% (5.5–10.4) vs. 4.8% (2.4–5.7), respectively; *p* < 0.001). They also had similar absolute 3-epi-25(OH)D levels (despite having lower 25(OH)D serum concentrations) than patients without cirrhosis. A progressive increase in relative 3-epi-25(OH)D levels was observed with more advanced cirrhosis (*p* < 0.001). An analysis of the ROC area under the curve determined 6% as the optimal cut-off for relative 3-epi-25(OH)D. All patients with Child–Pugh stage C and 88.6% with stage B were above the 6% cut-off and had significantly higher absolute serum 3-epi-25(OH)D concentrations (0.9 ng/mL vs. 0.6 ng/mL; *p* < 0.05) and lower serum 25(OH)D levels (9.3 vs. 14.1 ng/mL; *p* < 0.001) than patients <6% cut-off. **Conclusions**: These results reflect the marked increases in relative 3-epi-25(OH)D levels that occur with cirrhosis. The specific hepatic metabolic alterations still need to be unravelled, including whether cirrhosis might lead to reduced epimer clearance.

## 1. Introduction

Vitamin D deficiency frequently occurs in patients with liver diseases, given the liver’s central role in vitamin D metabolism [1]. In parallel to the standard 25-hydroxyvitamin D (25(OH)D) metabolic pathway, vitamin D can undergo hepatic conversion through a C-3 epimerisation pathway. This yields the C-3 epimer of 25-hydroxyvitamin (3-epi-25(OH)D) after reversal of the stereochemical configuration of the C-3-bound hydroxyl group (β→α). Immunoassays consistently measure the 3-epi-25(OH)D together with the status marker 25(OH)D due to their similar molecular weight and chemical structure and can overestimate vitamin D status. Advances in analytical methods, particularly high performance liquid chromatography tandem-mass spectrometry (LC-MS/MS), which is the gold standard, allow the accurate differentiation and quantification of 3-epi-25(OH)D from its precursor 25(OH)D [2,3].

In most healthy adults, absolute 3-epi-25(OH)D serum concentrations typically range from 0 to 26% (0.1–23.7 ng/mL) relative to 25(OH)D [4]. These are usually too low to contribute to significant overestimation of vitamin D status, although larger concentrations are observed and might depend on disease status [4,5,6,7,8,9,10,11]. The pathological relevance of the C-3 epimer remains unclear, however, it is increasingly associated with diseases such as diabetes, metabolic syndrome, arthritis and chronic kidney disease [12,13,14]. Specifically, increased 3-epi-25(OH)D has been suggested as a predictive biomarker for the progression and severity of chronic kidney disease [14].

Interestingly, infants, particularly when premature, consistently display higher serum 3-epi-25(OH)D concentrations (approximately 30% of 25(OH)D) [15]. This trend reverses during the first year to concentrations approximating 10% and this trajectory continues into adulthood [15]. Hepatic immaturity has been suggested to contribute to elevated serum 3-epi-25(OH)D levels in premature infants [11,16]. Indeed, liver dysfunction could plausibly alter these metabolic processes because the liver is the primary site for both vitamin D 25-hydroxylation [17] and epimerisation of 25(OH)D, driven by a yet-to-be-identified epimerase [18]. Even though the 3-epimerase enzyme has not been fully characterised, it is reported to be present in the microsomal fraction of liver cells, using nicotinamide adenine dinucleotide phosphate hydrogen (NADPH) as a cofactor. Tuckey et al. have shown that both rat and human 25-hydroxyvitamin D_3_ 3-epimerase catalyse the reversible interconversion of 25(OH)D_3_ and 3-epi-25(OH)D_3_ [18]. Moreover, an epidemiological study reported a positive correlation between the C-3 epimer and higher alcohol consumption, which can serve as a proxy for impaired liver function [3]. Thus, we postulated that altered vitamin D metabolism with higher epimerase activity might be detectable in patients with cirrhosis (impaired liver function). We previously quantified serum 3-epi-25(OH)D levels during supplementation with cholecalciferol (20,000 IU/week for 6 months) in 65 patients with chronic liver diseases [19]. Although no significant differences were observed between the 17 patients with cirrhosis and the 48 without, the small sample size may have influenced the findings. A recent study suggests that the proportion of 3-epi-25(OH)D relative to 25(OH)D (as opposed to absolute amounts) may offer a more meaningful indicator of the epimerase changes associated with various disease states [5].

The aim herein was to quantify 3-epi-25(OH)D levels in a larger cohort comprising patients with chronic liver diseases, including cirrhosis, to determine whether concentrations differ in patients with and without cirrhosis by comparing both absolute and relative amounts. The working hypothesis is that patients with cirrhosis have higher concentrations of 3-epi-25(OH)D due to impaired liver function.

## 2. Patients and Methods

### 2.1. Participants

Patients with chronic liver diseases (CLDs) were included from pre-existing cohorts originally recruited from Saarland University Medical Center (*n* = 212) and University Hospital Bonn (*n* = 97) in Germany. For a full explanation of these cohorts, please refer to previous publications [20,21]. All patients had a diagnosis of chronic liver disease, with variable aetiologies. The inclusion criteria were age > 18 years, a CLD diagnosis, and available serum for vitamin D analyses. Written informed consent was present for all patients, and ethical approval was provided by the local research ethics committees of Saarland University Medical Center (Ref. 57/11, 14 January 2014; 271/11, 23 January 2012), and University Hospital Bonn (Ref. 191/05, 23 January 2006). The study was carried out in accordance with the Declaration of Helsinki.

### 2.2. Serum Vitamin D Quantification

Vitamin D compounds were quantified from aliquoted serum samples (stored at −80 °C). The assay supported protein precipitation, liquid/liquid extraction of 75 μL of each sample, isotope internal standards for all vitamin D metabolites, and a one-pot double derivatisation scheme, which used acetylation after a Diels–Alder reaction to improve separation efficiency and provide baseline separations of the 3-epi-25(OH)D and 25(OH)D vitamin D metabolites in a conventional C-18 stationary phase. Liquid chromatography-tandem mass spectrometry (LC-MS/MS) analysis in single reaction monitoring mode of a Sciex QTRAP 6500+ mass spectrometer (Concord, ON, Canada) was implemented for mass spectral data acquisition. The full details of this method have been published elsewhere [22,23].

### 2.3. Quantification of Liver-Related Parameters

The diagnosis of cirrhosis was based on liver biopsy, liver stiffness measurement > 13 kPa by transient elastography (TE, Fibroscan; Echosens, Paris, France), and imaging (nodular appearance of liver with either splenomegaly, ascites, or varices) plus platelet count < 150/mL, as summarised in [24]. Clinical chemical assays were used to quantify liver function tests. Child–Pugh staging was used, which indicates severity of cirrhosis and includes five clinical and laboratory parameters: serum bilirubin, serum albumin, prothrombin time or INR, presence of ascites, and hepatic encephalopathy. A score of 1–3 is assigned to each parameter and the total score stages the patient as follows: Child–Pugh A (5–6 points, well-compensated cirrhosis with sufficient hepatic function); Child–Pugh B (7–9 points, significant functional compromise); and Child–Pugh C (10–15 points, decompensated cirrhosis). In addition, the Model for End-Stage Liver Disease (MELD) score was calculated, and this estimates the severity of chronic liver disease and is based on a formula including INR, serum bilirubin and creatinine levels. It is considered an objective measure of liver dysfunction with scores ranging from 6 (least severe) to 40 (most severe).

### 2.4. Statistical Analyses

Statistical analyses were carried out using SPSS 27.0 (IBM, Munich, Germany) and Graphpad Prism 10.6.1 (Graphpad Software Inc., San Diego, CA, USA), with a *p* < 0.05 threshold for statistical significance (two-sided tests). The primary aim was to compare absolute concentrations of 25(OH)D and 3-epi-25(OH)D, as well as relative amounts of 3-epi-25(OH)D in patients with and without cirrhosis and in different stages of cirrhosis (based on Child–Pugh staging and MELD score). The relative epimer contribution was calculated as the percentage of total 25(OH)D (defined as the sum of 25(OH)D and 3-epi-25(OH)D) [25]. The Kolmogorov–Smirnov test was used to assess the data distribution, which is presented as medians with interquartile ranges, and contingency tables (with Chi square test and Fisher’s exact test) assessed categorical data. Mann–Whitney and Kruskal–Wallis tests assessed for differences, e.g., age or vitamin D metabolites, liver function tests (LFTs) in patients stratified for cirrhosis and Child–Pugh stage and MELD score, while Bonferroni correction was used for pairwise comparisons. Correlations were assessed using Spearman’s correlation coefficient.

Patients were also grouped based on vitamin D status, in line with guideline recommendations and cut-offs consistently used in previous studies: sufficiency (≥30 ng/mL 25-hydroxyvitamin D), insufficiency (<30 and ≥20 ng/mL), deficiency (<20 and ≥10 ng/mL), and severe vitamin D deficiency (<10 ng/mL) [26]. Receiver operating characteristic (ROC) area under the curve (AUC) analysis determined the optimal cut-off for relative serum 3-epi-25(OH)D that is predictive of cirrhosis. Univariate and multivariate binary logistic regression analyses were conducted using relative 3-epi-25(OH)D cut-off of 6% (determined by AUC analysis) as a dependent variable, and included the following independent variables: serum 25(OH)D concentrations and age (as continuous variables), sex (binary variable), season (binary variable with summer/autumn vs. winter/spring), and the Child–Pugh stage (A/B/C/none). The MELD score was not included in the regression analysis due to missing values in 26% of patients with cirrhosis. Multivariate regression analysis was performed using the significant variables set at *p* < 0.01 in univariate analysis and a stepwise forward approach.

## 3. Results

### 3.1. Participant Characteristics

Of the 309 patients with CLD included, 82% (*n* = 254) had cirrhosis. The median age was 58 (51–66) years and 39% were women. Over half of the patients with cirrhosis were categorised as Child–Pugh stage A (*n* = 140, 55%), one third as Child–Pugh stage B (*n* = 79, 31%), and the remainder, who were suffering from more advanced cirrhosis, as Child–Pugh stage C (*n* = 35, 14%). The calculated MELD score was 11 (8–15), and significantly differed (*p* < 0.05) between the Child–Pugh stages: A (9, 7–11), B (14, 11–18) and C (19, 15–21).

The hepatitis C virus (HCV) infection was diagnosed in 27.5% of patients, whereas 7.1% had a chronic hepatitis B virus (HBV) infection. Alcohol-associated liver disease was diagnosed in 17.5%, non-alcoholic fatty liver disease (NAFLD; now referred to as metabolic dysfunction-associated steatotic liver disease (MASLD)) was diagnosed in 7.8%, and 5.8% had autoimmune hepatitis (AIH). The remaining 34.3% were grouped as “other” due to the heterogeneous nature and low percentage of other diagnoses, such as primary biliary cholangitis, primary sclerosing cholangitis, hereditary haemochromatosis, or cryptogenic aetiology. Table 1 summarises the main causes of CLD, stratified for cirrhosis. The majority of patients without cirrhosis had a diagnosis of viral hepatitis (HCV or HBV), followed by NAFLD, whereas patients with cirrhosis were mainly diagnosed with viral hepatitis C and alcohol-associated liver disease. Liver biochemistry differed significantly between groups, and patients with cirrhosis exhibited significantly higher serum aspartate aminotransferase (AST) levels, alkaline phosphatase (AP) levels, gamma-glutamyl transpeptidase (γ-GT) activities and total bilirubin values, and lower serum albumin levels (all *p* < 0.05).

### 3.2. Associations Among Vitamin D Metabolites

Absolute serum 3-epi-25(OH)D concentrations showed a strong positive correlation with 25(OH)D levels (Spearman’s correlation coefficient: 0.82, *p* < 0.001) in the overall cohort (as shown in Table 2). A weaker correlation was observed between relative and absolute 3-epi-25(OH)D concentrations (Spearman’s correlation coefficient: 0.27, *p* < 0.001). Additionally, serum 25(OH)D levels correlated inversely with relative 3-epi-25(OH)D (Spearman’s correlation coefficient: −0.28, *p* < 0.001). The distribution of absolute 3-epi-25(OH)D concentrations was significantly higher in autumn/summer as compared to blood sampling in winter/spring (1.0 (0.4–1.6) vs. 0.6 (0.2–1.1) ng/mL; *p* < 0.001). A similar seasonal variation was observed for serum 25(OH)D (13.8 (7.5–21.9) vs. 9.7 (4.1–17.4) ng/mL; *p* = 0.001). No consistent pattern was observed for relative 3-epi-25(OH)D, and this finding was further supported by regression analysis (described below).

**Figure 1:**
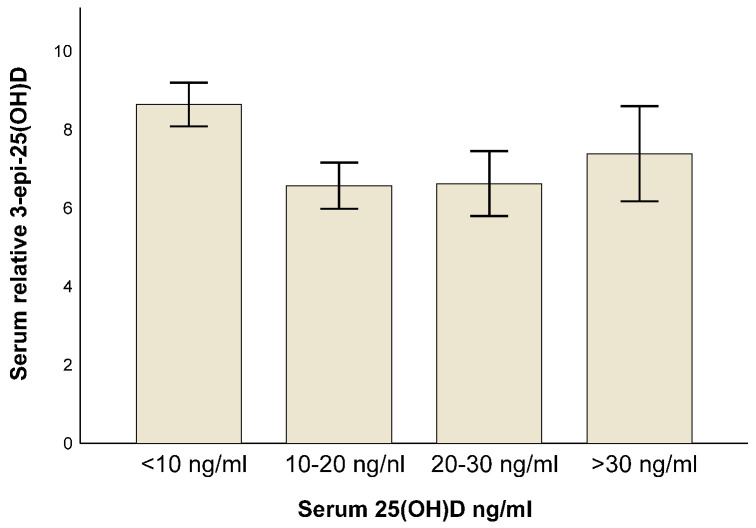
Histogram displaying relative 3-epi-25(OH)D in relation to serum 25(OH)D concentrations for the entire cohort of 309 patients with chronic liver diseases, as grouped based on accepted clinical cut-offs for vitamin D status. Error bars indicate 95% confidence intervals.

Patients were subsequently grouped based on established clinical cut-offs for vitamin D status, and these categories were compared to relative 3-epi-25(OH)D. We observed an increasing trend in relative 3-epi-25(OH)D levels with more sufficient vitamin D status; however, the overall pattern more closely resembled a U-shaped curve, with the highest relative 3-epi-25(OH)D levels detected in patients with severe vitamin D deficiency (<10 ng/mL; Figure 1).

### 3.3. Vitamin D Metabolites Stratified for Liver Cirrhosis and Severity

As shown in Table 2, serum 25(OH)D was positively correlated with 3-epi-25(OH)D in the overall cohort, with a stronger association observed in patients without cirrhosis. In this patient group, absolute and relative 3-epi-25(OH)D levels were also positively correlated, whereas this association was weaker in patients with cirrhosis. In addition, relative 3-epi-25(OH)D levels showed a positive correlation with 25(OH)D levels in patients without cirrhosis, but a negative correlation in patients with cirrhosis.

**Figure 2:**
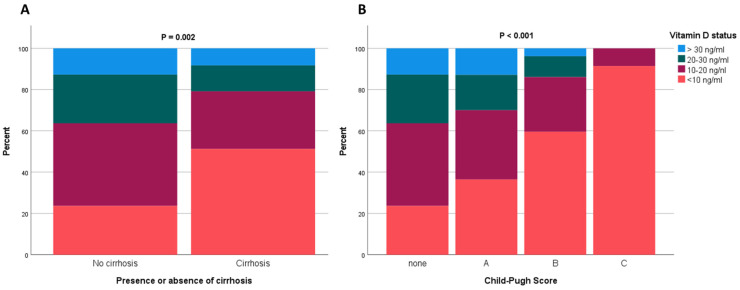
The proportion of patients in the different categories of vitamin D status are displayed based on (**A**) the presence or absence of cirrhosis and (**B**) Child–Pugh score. The Child-Pugh staging is defined as: Child–Pugh A (5–6 points, well-compensated cirrhosis with sufficient hepatic function); Child–Pugh B (7–9 points, significant functional compromise); and Child–Pugh C (10–15 points, decompensated cirrhosis).

In addition to these findings, patients with cirrhosis also had significantly lower serum 25(OH)D concentrations than those without cirrhosis: 9.7 ng/mL (4.1–18.0 ng/mL) vs. 15.3 ng/mL (9.7–23.5 ng/mL), respectively, (*p* < 0.001, Table 1). Despite declining 25(OH)D levels, absolute 3-epi-25(OH)D concentrations did not differ between these two subgroups. As shown in Figure 2A, the distribution of vitamin D status differed significantly between the groups (*p* = 0.002). The majority of patients without cirrhosis (40%) were categorised as deficient, in contrast to 51.2% of patients with cirrhosis who were severely vitamin D deficient. Stratification by Child–Pugh score demonstrated a progressive decline in vitamin D status with increasing disease severity (Figure 2B). Notably, severe vitamin D deficiency was more prevalent in patients with Child–Pugh stage C compared to patients with Child–Pugh stage A (91.4% vs. 36.4%, respectively; *p* < 0.001).

When stratified by the status of cirrhosis, Kruskal–Wallis tests revealed significant differences in both absolute and relative 3-epi-25(OH)D levels across categories of vitamin D status (all *p* < 0.001; Supplementary Table S1). Absolute 3-epi-25(OH)D concentrations decreased with declining vitamin D status across the entire cohort. However, values remained consistently higher in patients with cirrhosis than in those without, despite generally lower 25(OH)D concentrations across most categories. In patients without cirrhosis, relative C-3 epimer concentrations followed a similar pattern. In contrast, the opposite trend was observed in patients with cirrhosis, where relative 3-epi-25(OH)D levels increased with worsening vitamin D status. Specifically, within the severe vitamin D deficiency category, the relative 3-epi-25(OH)D was 2.4 (1.8–2.6) in patients without cirrhosis, versus 9.0 (6.2–12.1) in patients with cirrhosis (Supplementary Table S1).

**Figure 3:**
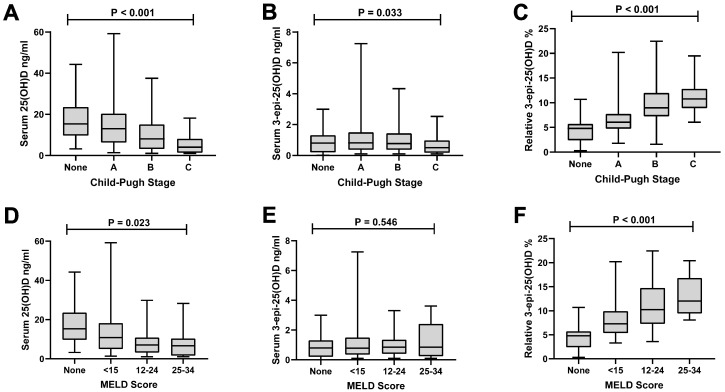
The following vitamin D metabolites are displayed for each Child–Pugh stage: (**A**) serum 25-hydroxyvitamin D (25(OH)D) concentrations, (**B**) serum 3-epi-25(OH)D concentrations, and (**C**) relative 3-epi-25(OH)D levels. Additionally, the vitamin D metabolites are displayed for each category of MELD scoring: (**D**) serum 25-hydroxyvitamin D (25(OH)D) concentrations, (**E**) serum 3-epi-25(OH)D concentrations, and (**F**) relative 3-epi-25(OH)D levels. Boxplots display median and minimum–maximum ranges. The 55 patients without cirrhosis were assigned “none” for Child–Pugh stage and MELD score. The Child-Pugh staging is defined as: Child–Pugh A (5–6 points, well-compensated cirrhosis with sufficient hepatic function); Child–Pugh B (7–9 points, significant functional compromise); and Child–Pugh C (10–15 points, decompensated cirrhosis).

With respect to disease severity, significant differences in median 25(OH)D and absolute 3-epi-25(OH)D concentrations were observed across Child–Pugh stages (*p* < 0.001 and *p* = 0.033, respectively), with 25(OH)D levels decreasing as the Child–Pugh stage increased and absolute 3-epi-25(OH)D levels declining slightly (Figure 3A,B). In contrast, relative 3-epi-25(OH)D levels increased significantly (*p* < 0.001) with the advancing disease (Figure 3C). Pairwise comparisons demonstrated significant differences in relative 3-epi-25(OH)D levels between most groups (all *p* < 0.001, except for Child–Pugh B versus C, adjusted *p* = 0.447). Similar associations were observed when the data were stratified based on MELD results; however, no significant differences in absolute 3-epi-25(OH)D concentrations were observed, and these remained relatively stable despite decreases in serum 25(OH)D levels with higher MELD scores (Figure 3D–F).

### 3.4. AUC and Regression Analysis

**Figure 4:**
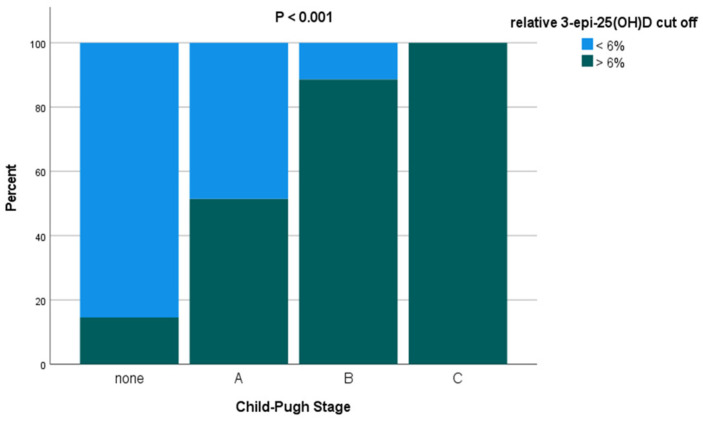
The area under the ROC curve analysis identified the optimal cut-off for relative 3-epi-25(OH)D level in serum with the maximum sensitivity and specificity as being 6%. The Child-Pugh staging is defined as: Child–Pugh A (5–6 points, well-compensated cirrhosis with sufficient hepatic function); Child–Pugh B (7–9 points, significant functional compromise); and Child–Pugh C (10–15 points, decompensated cirrhosis).

The AUC analysis identified the relative 3-epi-25(OH)D levels in serum as a significant predictor of cirrhosis in this cohort (AUC = 0.84; 95% CI 0.79–0.84; and *p* < 0.001). The optimal cut-off for distinguishing patients with and without cirrhosis using relative 3-epi-25(OH)D levels with maximum sensitivity and specificity was 6% (group characteristics based on this cut-off are summarised in Table 3). In brief, all patients with advanced cirrhosis, as indicated by Child–Pugh stage C, had relative 3-epi-25(OH)D levels ≥ 6%, as compared to 70 patients (88.6%) with Child–Pugh B and 72 patients (51.4%) with Child–Pugh A (Figure 4). Only 8 patients (14.5%) without cirrhosis displayed relative 3-epi-25(OH)D levels ≥ 6% (all *p* < 0.001). Of note, the AUC performed better for relative 3-epi-25(OH)D levels than for serum 25(OH)D levels as a predictor of cirrhosis (AUC = 0.66; 95% CI 0.59–0.73; and *p* < 0.001).

When comparing patient characteristics, significantly more men and patients with alcohol-associated liver disease presented with relative 3-epi-25(OH)D levels ≥ 6% (both *p* < 0.0001). Patients above this cut-off also had significantly lower serum albumin concentrations and significantly higher LFTs (apart from alanine aminotransferase (ALT); Table 3), indicating a more advanced disease. They also had significantly higher absolute serum 3-epi-25(OH)D concentrations than patients below this cut-off (0.9 ng/mL vs. 0.6 ng/mL and *p* < 0.05) and lower serum 25(OH)D levels (9.3 vs. 14.1 ng/mL; *p* < 0.001). Moreover, significantly (*p* < 0.0001) more patients with ≥6% relative C-3 epimer had 25(OH)D < 10 ng/mL as compared to patients with <6%: 106 (74.1%) vs. 37 (25.9%), respectively.

In univariate analysis, serum 25(OH)D and Child–Pugh stages were predictors of higher relative 3-epi-25(OH)D concentrations (≥6% cut-off). Age, sex and season were not associated with this threshold (Table 4). In multivariate analysis, only the Child–Pugh stage remained a predictor of a relative 3-epi-25(OH)D level ≥ 6% (odds ratio [OR] = 7.05; 95% confidence interval [CI], 4.29–11.58; and *p* < 0.001).

## 4. Discussion

We hypothesised that cirrhosis (impaired hepatic function) may alter vitamin D metabolism with upregulated hepatic C-3 epimerisation, as seen in premature infants, who have elevated serum 3-epi-25(OH)D [16]. We observed significantly increased relative 3-epi-25(OH)D levels in patients with cirrhosis when compared to patients without cirrhosis. An increase in relative 3-epi-25(OH)D was observed despite low substrate concentrations of serum 25(OH)D and was more pronounced based on the severity of the cirrhosis, as assessed using Child–Pugh staging and MELD scoring systems.

Serum vitamin D levels declined with increasing Child–Pugh stage, with 91% of patients at stage C classified as severely deficient versus 36% at stage A [27]. Patients with advanced cirrhosis displayed significantly higher relative 3-epi-25(OH)D when compared to patients with a less advanced stage of the disease, despite the reduced availability of the 25(OH)D substrate. This observation partially supports findings by Chen et al. [5], who reported consistent changes in both the absolute and relative 3-epi-25(OH)D levels across various diseases. Specifically, they observed decreased absolute serum 3-epi-25(OH)D levels in 8 of 11 disease categories (which included 32 specific diseases), while relative 3-epi-25(OH)D levels increased, comparable to our findings. However, the relative C-3 epimer values observed by Chen et al. [5] were lower than those observed in our patients with cirrhosis and resembled the values we observed in patients without cirrhosis (ca. 4–5%). This may be partially because Chen et al. only included autoimmune hepatitis among liver diseases.

Absolute 3-epi-25(OH)D concentrations in our cohort were comparable to those reported in the literature and did not differ significantly when comparing patients with cirrhosis to those without, despite significantly lower 25(OH)D in patients with cirrhosis [5,6,28]. Given the lower substrate availability, it is unclear whether absolute 3-epi-25(OH)D levels remain or appear stable, given the lower substrate availability. This finding may reflect the suggestion by Chen et al. that the relative 3-epi-25(OH)D level may better reflect pathological epimer upregulation in disease-specific contexts, as it partially accounts for differences in the level of its substrate 25(OH)D, and may reduce, potential confounding [5]. It is, however, not a direct measure of epimerisation activity. We observed absolute epimer values to remain relatively stable in patients with cirrhosis at Child–Pugh stages A and B, but to be reduced in Child–Pugh C stage. This finding might be influenced by the sample sizes because, when stratifying for MELD score, we observed absolute serum 3-epi-25(OH)D to remain stable regardless of the significant decreases in 25(OH)D levels with advancing MELD score. Likewise, as with Child–Pugh staging, relative 3-epi-25(OH)D levels significantly increased with advancing MELD. These findings might be an indication of impaired vitamin D metabolism, with reduced clearance of the 3-epi-25(OH)D in relation to 25(OH)D in the setting of cirrhosis.

In a pooled analysis of 3610 samples, Kubiak et al. [6] observed strong positive correlations between serum 25(OH)D and absolute 3-epi-25(OH)D levels and an S-shaped increase in the relative proportion of 3-epi-25(OH)D across 25(OH)D categories with higher proportions of 3-epi-25(OH)D observed at higher 25(OH)D levels; moreover, serum 25(OH)D was a significant predictor of absolute 3-epi-25(OH)D levels in linear regression analysis. They observed that only 2% of patients with low serum 25(OH)D concentrations (e.g., <20 ng/mL) had relative 3-epi-25(OH)D levels > 10%. In contrast, when comparing our cohort in this way, we observed that 31% of 201 patients with cirrhosis and a serum 25(OH)D level < 20 ng/mL had relative 3-epi-25(OH)D levels > 10%. Of note, only one patient without cirrhosis presented with relative 3-epi-25(OH)D levels > 10%. Owing to the smaller sample size in the present study, patients were grouped based on established clinical cut-offs for vitamin D status. Although we observed an increasing trend in relative 3-epi-25(OH)D level with more sufficient vitamin D status, the overall pattern more closely resembled a U-shaped curve, with the highest relative 3-epi-25(OH)D levels detected in patients with severe vitamin D deficiency. We observed a positive correlation between relative 3-epi-25(OH)D and 25(OH)D levels in patients without cirrhosis, but it was negatively correlated in patients with cirrhosis. These opposing patterns might represent different segments of the U-shaped curve.

The AUC analysis identified the relative 3-epi-25(OH)D level as a strong predictor of advanced cirrhosis when using a 6% cut-off. Specifically, all patients with Child–Pugh stage C and the majority of patients with Child–Pugh stage B (corresponding to advanced cirrhosis) were above this cut-off. In contrast, only half of patients with Child–Pugh A and 15% of patients without cirrhosis were >6% cut-off. Moreover, significantly higher LFT values were demonstrated in the patients with relative 3-epi-25(OH)D levels ≥ 6% as compared to <6%, indicating greater liver injury. Interestingly, these patients also exhibited higher absolute 3-epi-25(OH)D levels and lower 25(OH)D levels in serum. Binary logistic regression corroborated these findings where the Child–Pugh stage was independently associated with relative 3-epi-25(OH)D levels using the 6% cut-off.

Genetic studies confirm CYP2R1 in hepatic 25-hydroxylation, but not in C-3 epimer formation [29] indicating that another enzyme is responsible for C-3 epimerisation [30]. Indeed, *CYP2R1* variants (rs2060793) have been significantly associated with 25(OH)D levels, whereas 3-epi-25(OH)D correlated with *DHCR7/NADSYN1* (rs12785878) and *GC* (rs2282679) polymorphisms [30]. Accordingly, the metabolism of the epimer remains incompletely understood, and it is unknown whether other vitamin D metabolites can act as substrate for C-3 epimer formation. As such, the contribution of potential extrahepatic sites of vitamin D metabolism may have influenced the findings. Moreover, a healthy population control group would have been advantageous to include as would a larger sample size of patients with chronic liver diseases and no cirrhosis and patients at Child–Pugh stage C. Despite most patients displaying low serum 25(OH)D levels, a lack of data on sun exposure, vitamin D supplementation or the presence of other comorbidities such as renal dysfunction might have also affected our results. The precise measurement of serum 25(OH)D and 3-epi-25(OH)D levels, using gold standard LC-MS/MS optimised for the C-3 epimer, is, however, a key strength.

Recent vitamin D guidelines published by the Endocrine Society [31] acknowledge the need for large clinical trials and biomarkers to flag high-risk individuals and predict disease risk. The C-3 epimer might have potential as a biomarker for advanced liver injury and cirrhosis, as it does for advanced kidney disease. Moreover, further exploration of the epimer in the pre-cirrhosis setting is warranted, given that significant differences in relative epimer concentrations were observed between patients with no cirrhosis and those at Child–Pugh stage A. Therefore, a comparison of patients with different degrees of fibrosis might shed more light on epimer behaviour in liver disease before the disease reaches a more advanced stage.

## 5. Conclusions

The results demonstrate marked increases in relative 3-epi-25(OH)D levels in patients with cirrhosis. This increase in relative C-3 epimer occurred despite low 25(OH)D substrate concentrations. Because the metabolic alteration leading to increased epimer concentrations reported in infants has been attributed to hepatic immaturity and is possibly a protective mechanism, we speculate that cell plasticity in cirrhosis might lead to alterations in the hydroxylation process with possible reduced epimer clearance. Thus, the role of C-3 epimerisation in cirrhosis requires further investigation, including longitudinal validation. Unravelling the mechanistic effects will help shed light on the novel molecular processes regulating hepatic vitamin D metabolism.

## Figures and Tables

**Table 1:** Baseline patient characteristics based on presence of cirrhosis.

	No Cirrhosis	Cirrhosis
**Sociodemographic**		
N (male/female)	55 (25/30)	254 (165/89) *
Age (years)	55 (49–61)	59 (52–67) *
**Cause of chronic liver disease *n* (%)**	55 (100)	254 (100) ** ^§^
Alcoholic liver disease	1 (1.8)	53 (20.9)
Hepatitis C virus (HCV)	20 (36.4)	65 (26.6)
Hepatitis B virus (HBV)	13 (23.6)	9 (3.5)
NAFLD (MASLD)	9 (16.4)	15 (5.9)
Autoimmune hepatitis	7 (12.7)	11 (4.3)
Other	5 (9.1)	101 (39.8)
**Liver function tests**		
ALT (U/L)	34 (25–67)	40 (25–75)
AST (U/L)	33 (27–52)	54 (36–88) **
γ-GT (U/L)	47.5 (24.0–89.0)	142.5 (70.3–310.5) **
AP (U/L)	75 (61–97)	114 (84–169) **
Total bilirubin (mg/dL)	0.6 (0.4–0.8)	1.1 (0.7–2.4) **
Albumin (g/L)	46 (44–48)	36.1 (29.0–42.0) **
**Vitamin D metabolites**		
25(OH)D (ng/mL)	15.3 (9.7–23.5)	9.7 (4.1–18.0) **
Total 25(OH)D (ng/mL)	16.2 (10.0–24.3)	10.7 (4.6–19.3) **
3-epi-25(OH)D (ng/mL)	0.8 (0.2–1.3)	0.8 (0.4–1.4)
Relative 3-epi-25(OH)D (%)	4.8 (2.4–5.7)	7.4 (5.5–10.4) **

Values presented as median (interquartile range). Significant differences between groups denoted by * *p* < 0.05 and ** *p* < 0.001. ^§^ Differences across categories of causes of chronic liver disease differed significantly between patients with and without cirrhosis. Abbreviations: ALT, alanine aminotransferase; AP, alkaline phosphatase; AST, aspartate aminotransferase; γ-GT, gamma-glutamyl transpeptidase; NAFLD, non-alcoholic fatty liver disease; and MASLD, metabolic dysfunction-associated steatotic liver disease.

**Table 2:** Correlations between vitamin D metabolites.

	25(OH)D	3-epi-25(OH)D	Relative 3-epi-25(OH)D
25(OH)D		0.82	−0.28
3-epi-25(OH)D	0.82		0.27
relative 3-epi-25(OH)D	−0.28	0.27	
**Cirrhosis**			
25(OH)D		0.86	−0.30
3-epi-25(OH)D	0.86		0.20
relative 3-epi-25(OH)D	−0.30	0.20	
**No cirrhosis**			
25(OH)D		0.91	0.60
3-epi-25(OH)D	0.91		0.86
relative 3-epi-25(OH)D	0.60	0.86	
The data show Spearman’s correlation coefficient rho			
All correlations were significant (*p* < 0.01).			

**Table 3:** Baseline characteristics of patients with cirrhosis based on relative C-3 epimer cut-off status.

	C-3 Epimer Cut-Off < 6%	C-3 Epimer Cut-Off ≥ 6%
**Sociodemographic**		
N (male/female)	124 (70/54)	185 (120/65) ***
Age (years)	58 (51–67)	58 (51–66)
**Cause of chronic liver disease *n* (%)**	124 (100)	185 (100) *** ^§^
Alcoholic liver disease	5 (4.0)	49 (26.5)
Hepatitis C virus (HCV)	47 (37.9)	38 (20.5)
Hepatitis B virus (HBV)	17 (13.7)	5 (2.8)
NAFLD (MASLD)	13 (10.5)	11 (5.9)
Autoimmune hepatitis	6 (4.8)	12 (6.5)
Other	36 (29.1)	70 (37.8)
**Liver function tests**		
ALT (U/L)	39.0 (26.0–70.0)	40.0 (25.0–76.0)
AST (U/L)	39.5 (29.0–60.0)	60.5 (39.0–96.5) **
γ-GT (U/L)	85.0 (40.0–150.0)	158.5 (74.0–348.0) **
AP (U/L)	82.0 (69.0–109.0)	125.0 (97.0–201.0) **
Total bilirubin (mg/dL)	0.6 (0.5–1.0)	1.4 (0.8–3.3) **
Albumin (g/L)	44.0 (40.0–47.0)	33.0 (27.0–39.0) ***
**Vitamin D metabolites**		
25(OH)D (ng/mL)	13.5 (8.0–21.6)	8.6 (3.3–17.0) **
Total 25(OH)D (ng/mL)	14.1 (8.5–22.6)	9.3 (3.8–18.6) **
3-epi-25(OH)D (ng/mL)	0.6 (0.3–1.1)	0.9 (0.4–1.6) *
Relative 3-epi-25(OH)D (%)	4.7 (3.6–5.4)	9.0 (7.3–11.7) ***

Values presented as median (interquartile range). Significant differences between groups denoted by * *p* < 0.05, ** *p* < 0.001, and *** *p* < 0.0001. ^§^ Differences across categories of causes of chronic liver disease differed significantly between patients with and without cirrhosis. Abbreviations: ALT, alanine aminotransferase; AP, alkaline phosphatase; AST, aspartate aminotransferase; γ-GT, gamma-glutamyl transpeptidase; NAFLD, non-alcoholic fatty liver disease; and MASLD, metabolic-dysfunction associated steatotic liver disease.

**Table 4:** Univariate and multivariate binary logistic regression analysis of predictors of relative 3-epi-25(OH)D serum concentrations.

**(A) Univariate Analysis**			
**Factor**	**OR**	**95% CI**	***p***
25(OH)D ng/mL	0.95	0.93–0.98	<0.001
Child–Pugh stage	7.36	4.52–12.00	<0.001
Age	1.00	0.98–1.02	0.829
Sex (male vs. female)	0.70	0.44–1.12	0.14
Season	1.17	0.70–1.97	0.56
**(B) Multivariate analysis**			
**Factor**	**OR**	**95% CI**	***p***
25(OH)D ng/mL	0.99	0.96–1.01	0.35
Child–Pugh stage	7.05	4.29–11.58	<0.001

Abbreviations: CI, confidence interval; OR, odds ratio; 25(OH)D, 25-hydroxyvitamin D; and 3-epi-25(OH)D, 3-epi-25-hydroxyvitamin D.

## Data Availability

The original contributions presented in this study are included in the article/Supplementary Material. Further inquiries can be directed to the corresponding author.

## Supplementary Materials

The following supporting information can be downloaded at: https://www.mdpi.com/article/10.3390/nu18071071/s1, Table S1: Serum 25(OH)D and C-3 epimer values based on vitamin D status.
