# Supplementary material for: Elevated Relative Levels of the C-3 Epimer of 25-Hydroxyvitamin D in Patients with Cirrhosis
Source: Nutrients. 2026 Mar 27;18(7):1071. doi: 10.3390/nu18071071 (PMC13074843; doi:10.3390/nu18071071)
Supplement: Supplementary file 1 [file nutrients-18-01071-s001.zip › nutrients-3954706-supplementary.pdf]

Supplementary Table S1. Serum 25(OH)D and C-3 epimer values based on vitamin D status

	**Sufficient (> 30 ng/ml)**	**Insufficient (20 – 30 ng/ml)**	**Deficient (10 – 20 ng/ml)**	**Severely deficient (< 10 ng/ml)**
Patients without cirrhosis	7	13	22	13
25(OH)D (ng/ml)	39.2 (33.0 – 40.9)	23.5 (21.6 – 26.7)	12.2 (10.1 – 16.4)	7.2 (5.5 – 8.1)
3-epi-25(OH)D (ng/ml)	2.4 (2.2 – 2.7)	1.3 (1.0 – 1.5)	0.6 (0.3 – 0.9)	0.2 (0.1 – 0.2)
Relative 3-epi-25(OH)D (%)	5.8 (5.5 – 8.2)	5.2 (4.4 – 5.7)	4.5 (2.5 – 5.6)	2.4 (1.8 – 2.6)
Patients with cirrhosis	21	32	71	130
25(OH)D (ng/ml)	35.6 (31.4 – 38.7)	23.9 (20.4 – 27.0)	13.4 (11.3 – 17.4)	4.4 (2.4 – 7.1)
3-epi-25(OH)D (ng/ml)	2.8 (2.0 – 3.7)	1.7 (1.2 – 2.2)	1.0 (0.7 – 1.3)	0.4 (0.2 – 0.6)
Relative 3-epi-25(OH)D (%)	6.0 (5.4 – 9.0)	6.9 (4.7 – 8.2)	6.6 (4.9 – 8.4)	9.0 (6.2 – 12.1)

Kruskal-Wallis tests demonstrated significant differences (all $P < 0.001$) in absolute and relative 3-epi-25(OH)D concentrations as well as in serum 25(OH)D levels in patients with cirrhosis when grouped based on categories of vitamin D status and in patients without cirrhosis when grouped the same way.
